# Catalytic Differences between Flavohemoglobins of *Giardia intestinalis* and *E. coli*

**DOI:** 10.3390/pathogens13060480

**Published:** 2024-06-06

**Authors:** Sarah Hill, Isabelle Decorso, Novin Nezamololama, Zahra Babaei, Steven Patrick Rafferty

**Affiliations:** 1 Environmental and Life Sciences Graduate Program, Trent University, Peterborough, ON K9J 7B8, Canada; sarahhill2@trentu.ca (S.H.); isabelledecorso@trentu.ca (I.D.); novinnezamoldama@trentu.ca (N.N.); 2 Department of Parasitology & Mycology, Kerman University of Medical Sciences, Kerman 76169-13555, Iran; bzahra580@gmail.com

**Keywords:** Giardia, flavohemoglobin, nitric oxide, oxidative stress

## Abstract

The sole known heme enzyme of the parasitic protist *Giardia intestinalis* is a flavohemoglobin (gFlHb) that acts as a nitric oxide dioxygenase (NOD) and protects the organism from the free radical nitric oxide. To learn more about the properties of this enzyme, we measured its nitric oxide dioxygenase, NADH oxidase, and cytochrome *c* reductase activities and compared these to the activities of the *E. coli* flavohemoglobin (Hmp). The turnover number for the NOD activity of gFlHb (23 s^−1^) is about two-thirds of that of Hmp (34 s^−1^) at pH 6.5 and 37 °C. The two enzymes differ in their sensitivity towards molecules that act as heme ligands. For both gFlHb and Hmp, inhibition with miconazole, a large imidazole ligand, is adequately described by simple competitive inhibition, with *K*_I_ = 10 μM and 0.27 μM for gFlHb and Hmp, respectively. Inhibition plots with the small ligand imidazole were biphasic, which is consistent with previous experiments with carbon monoxide as a probe that show that the active site of flavohemoglobins exists in two conformations. Interestingly, the largest difference is observed with nitrite, which, like imidazole, also shows a biphasic inhibition plot; however, nitrite inhibits gFlHb at sub-millimolar concentrations while Hmp is not significantly affected. NADH oxidase activity measured under aerobic conditions in the absence of nitric oxide for Hmp was more than twice the activity of gFlHb. The addition of 1 mM hydrogen peroxide in these assays stimulated the NADH oxidase activity of gFlHb but not Hmp. Both enzymes had nearly identical cytochrome *c* reductase activities but the extent of the contribution of indirect reduction by flavohemoglobin-generated superoxide was much lower with gFlHb (4% SOD-inhibited) than with Hmp (17% SOD-inhibited). Although the active sites of the two enzymes share the same highly conserved residues that are important for catalysis, differences in the distal ligand binding site may account for these differences in activity and sensitivity towards NOD inhibitors. The differences observed in the NADH oxidase and cytochrome *c* reductase assays suggest that gFlHb may have evolved to protect the protist, which lacks both superoxide dismutase and catalase, from the damaging effects of superoxide by minimizing its production and from peroxide by actively reducing it.

## 1. Introduction

The parasitic protist *Giardia intestinalis* possesses flavohemoglobin, which is its sole known heme enzyme. All flavohemoglobins share a bidomain structure in which an amino-terminal, heme-binding globin domain is fused to a ferredoxin nucleotide reductase (FNR) domain that binds the cofactor flavin adenine dinucleotide (FAD) and the two-electron donor nicotinamide adenine dinucleotide (NADH) [1]. Flavohemoglobins have nitric oxide dioxygenase (NOD) activity. During the catalytic cycle molecular oxygen binds to ferrous heme which oxidizes NO to nitrate (NO_3_^−^), leaving the heme in the ferric state; an electron, ultimately provided by NADH and delivered via FAD, restores heme to the ferrous state [2]. Flavohemoglobins are also found in bacteria, yeast, and other protists such as Dictyostelium [3,4]. The most well-studied example is the *E. coli* flavohemoglobin Hmp [5]. Like Hmp, Giardia flavohemoglobin (gFlHb) has NOD activity and its expression is induced in response to nitrosative stress [6,7]. The sequence identity of gFlHb is higher towards *E. coli* (42%) than either Dictyostelium or Saccharomyces (33%), which suggests that Giardia may have acquired it by lateral gene transfer from a Gram-negative bacterial source. At 52 kDa, gFlHb is larger than any reported flavohemoglobin (~44 kDa), mainly due to the presence of two sequence inserts, one in the globin domain (residues 71–91) and the other in the FNR domain (residues 281-–307). The molecular modelling of gFlHb with both Alphafold and Swiss Model predicts that these two inserts interact with each other and increase the contact surface between the domains. As oxygen activation rather than reversible oxygen binding is required of the heme, the heme binding site of the globin domain contains a glutamic acid residue (H23) that hydrogen bonds to the proximal histidine ligand (F8). On the distal oxygen-binding face of the heme, flavohemoglobins have a tyrosine (B10), glutamine (E7), and leucine (E11) rather than a distal histidine residue at E7 as is found in myoglobin and mammalian hemoglobin [8,9]. These residues are conserved among flavohemoglobins, but the heme active site still has positions at which sequence variation is observed that may lead to altered enzymatic properties within members of this protein family, as has been observed with compounds that act as inhibitors of nitric oxide dioxygenase (NOD) activity [10]. We were especially interested in comparing the properties of gFlHb to Hmp as both organisms inhabit the gastrointestinal tract, although at different locations within. To this end, we expressed both enzymes and compared their NOD activities, NADH oxidase activities, and cytochrome *c* reductase activities. The influence of other agents on these activities was also compared.

## 2. Procedures

### 2.1. Protein Expression

gFlHb (Assemblage A, strain WB) and Hmp were expressed as recombinant proteins bearing an N-terminal His_6_-tag from a pET14b-based vector in BL21(DE3). Expression and purification conditions were as described previously [6], with the exception that cultures were autoinduced from Luria Broth media containing 0.5% glycerol, 0.05% glucose, and 0.2% lactose rather than using isopropyl thiogalactoside. In addition, 100 μM of the heme precursor 5-aminolevulinic acid was included in the media. As a final purification step, size exclusion chromatography on a Superdex 200 10/300 GL column (Cytiva Life Sciences, Vancouver, BC, Canada) was performed after immobilized metal affinity chromatography on NiNTA-Sepharose (Fisher Scientific, Ottawa, ON, Canada). The UV-visible spectrum of purified flavohemoglobins was recorded between 250 and 700 nm to ensure that residual imidazole, which can act as a heme ligand, had been removed. Purified enzymes were stored at −20 °C in buffer containing 50% glycerol.

### 2.2. Nitric Oxide Dioxygenase Assays

To determine NOD activity, Michaelis constant, and enzyme inhibition, rate assays were performed on a World Precision Instruments free radical analyzer (WPI, Sarasota, FL, USA) equipped with an NO-sensitive electrode and a temperature probe, connected to an analog digital converter controlled by Labscribe software, version 4 (iWorx, Dover, NH, USA). Before each set of experiments, the instrument was calibrated by adding aliquots of a 10.0 mM sodium nitrite standard ((MilliporeSigma, Oakville, ON, Canada) to a solution of 0.1 M KI in 0.1 M H_2_SO_4_, and measuring the current response, according to the manufacturer’s instructions. A linear response was observed with slopes of 2.3 to 2.8 nA/μM NO.

Assays were conducted on a 6 mL scale in cropped 50 mL Falcon tubes in a buffer containing 0.1 M Bis-Tris (pH 6.5) and 100 μM NADH. To minimize protein adhesion to the walls of the chamber, 0.25 mg/mL bovine serum albumin (BSA; Fisher Scientific, Ottawa, ON, Canada) was added. The presence of BSA had no effect on the observed NOD activities. Reactions were run at 37 °C in a stirred cell block heater with a four-placed machined aluminum block that matched the taper of the Falcon tubes. With this block arrangement up to four assay solutions could be warmed to the desired temperature beforehand. Upon thermal equilibration to 37 °C, the NO donor compound PROLI-NONOate (Cayman Chemical, Ann Arbor, MI, USA) prepared in 10 mM NaOH was added; 2 moles of NO are released per mole of donor. At the peak of NO release, which occurred within several seconds, flavohemoglobin and FAD were added to a final concentration of 2 nM and 10 nM, respectively. NOD activity was measured as the initial rate of NO signal decrease over the first 15 to 30 s of the reaction. For gFlHb, the Michaelis constant towards NO (*K*_m,NO_) was determined by holding the NADH concentration constant at 100 μM and varying the concentration of NO up to 3 μM; the Michaelis constant towards NADH (*K*_m,NADH_) was determined by holding the NO concentration constant at 2 μM and varying the NADH concentration up to 200 μM. Rate data were fitted to the Michaelis–Menten equation by nonlinear regression using Graphpad Prism software, version 9.4.1 (Dotmatics, Boston MA, USA).

**Figure 1:**
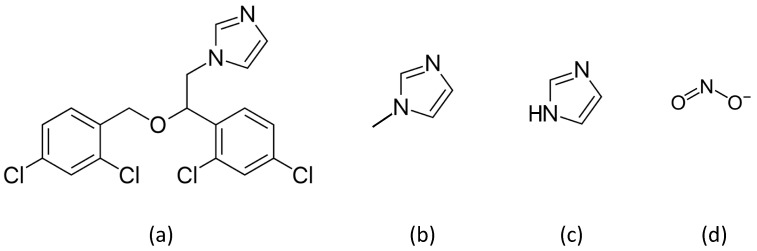
Compounds used as nitric oxide dioxygenase inhibitors: (**a**) miconazole; (**b**) 1-methylimidazole; (**c**) imidazole; and (**d**) nitrite.

**Figure 2:**
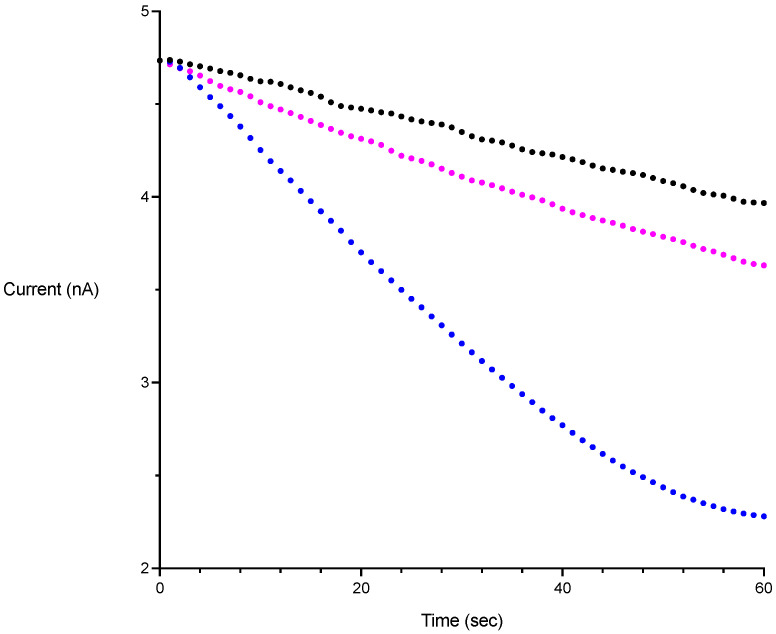
Sample rate measurements obtained on a free radical analyzer equipped with an NO sensitive electrode. Change in current is proportional to change in [NO]. PROLI-NONOate was added to a final concentration corresponding to 1 μM NO. Black trace: uncatalyzed. Blue trace: with 2 nM gFlHb. Purple trace: 2 nM gFlHb and 20 μM miconazole.

NOD inhibition assays for gFlHb and Hmp were performed as described above in buffer containing 100 μM NADH and 1 μM NO. The inhibitor was included in the assay solution before the addition of PROLI-NONOate. Inhibitors were also preincubated in the enzyme stock solutions at the same concentration. For each inhibitor, experiments with gFlHb and Hmp were run on the same day. All inhibition experiments were done using the same enzyme preparations. Each assay at a particular concentration of inhibitor was measured in triplicate or more. Four compounds were tested for their ability to act as inhibitors of NOD activity (Figure 1): three of these contain the imidazole functional group (miconazole, 1-methylimidazole, and imidazole); nitrite (NO_2_^−^) was the remaining compound tested. To account for the presence of the carrier solvent DMSO in experiments done with miconazole, solvent control experiments were run containing 0.34% DMSO which was maintained for all concentrations of miconazole tested. A set of sample kinetic runs is shown in Figure 2. To determine inhibition constants *K*_i_, rate data obtained at different inhibitor concentrations were fit to the equation for non-competitive inhibition nonlinear regression (Equation (1)) using Graphpad Prism software.

(1) $$v=\frac{V_{max}}{(K_{m,NO}+[NO])\left(1+\frac{\left[I\right]}{K_{i}}\right)}$$

### 2.3. Optical Titrations

Titrations of ferric gFlHb and Hmp were performed in 0.1 M Bis-Tris buffer (BioShop, Burlington, ON, Canada) pH 6.5 on a Cary 400 Bio UV-visible spectrophotometer (Agilent Technologies, Mississauga, ON, Canada). A series of solutions with 3.5 μM flavohemoglobin and nitrite concentrations ranging from 0 to 750 μM were prepared and their spectra measured between 700 and 250 nm. The binding constant *K*_D_ was determined from plots of the change in absorbance in the Soret region of the difference spectra as a function of nitrite concentration.

### 2.4. NADH Oxidase Assays

Rate measurements were performed using a Shimadzu UV-1900i UV-visible spectrophotometer (Shimadzu Corporation, Kyoto, Japan). Assays were performed in 0.1 M Bis-Tris buffer, pH 6.5, containing 0.10 μM flavohemoglobin and 1 μM FAD at 37 °C. Reactions were initiated by the addition of a 10 mM NADH stock solution to a final concentration of 100 μM NADH, and the absorbance decrease was monitored at 340 nm for one minute. Absorbance values were reported in terms of specific activity, corresponding to the number of moles of NADH oxidized per mole of enzyme (ε_340_ = 6.22 mM^−1^cm^−1^). For each condition, reactions were performed in triplicate. In certain cases, flavohemoglobin inhibitors were introduced at concentrations varying from 0 μM to 1000 μM, based on their binding affinities for the flavohemoglobin.

### 2.5. Cytochrome c Reductase Assays

Reduction assays were performed as described for the NADH oxidase assays with the following modifications. Instead of 0.10 μM flavohemoglobin, 0.05 μM flavohemoglobin was used. A total of 25 μM cytochrome *c* was included in the assay solution, and measurements followed the increase in cytochrome *c* reduction by the absorbance increase at 550 nm. Absorbance values were reported in terms of specific activity, corresponding to the number of moles of cytochrome *c* reduced per enzyme (ε_550_ = 22.1 mM^−1^cm^−1^). In certain cases, 5 U/mL CuZn SOD (Worthington Biochemicals, Lakewood, NJ, USA) was included in assays to determine the contribution of superoxide to the rate of cytochrome *c* reduction.

## 3. Results and Discussion

**Figure 3:**
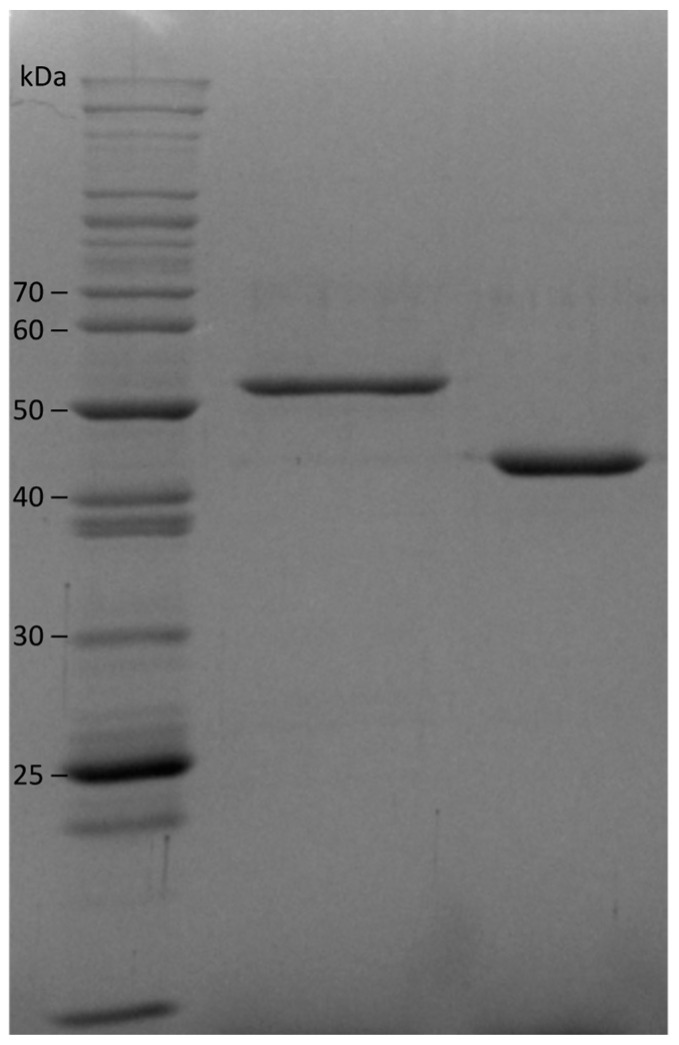
SDS-PAGE of purified recombinant flavohemoglobins. Lane 1, size ladder. Lane 2, Giardia flavohemoglobin gFlHb (54.4 kDa). Lane 3, *E. coli* Hmp (46.3 kDa).

Our expression conditions were similar to what we have used previously but with three modifications. We added 5-aminolevulinic acid (ALA) to the media to stimulate heme production, as this is the first committed heme precursor and it induces this biosynthetic pathway. Instead of using induction with IPTG, we used autoinduction with a mixture of glucose, lactose, and glycerol in the media [11]. Glucose is metabolized first, and cell mass increases; at the point where glucose has been consumed, the cells switch to metabolizing lactose and induce the lac operon as well as the expression of recombinant proteins under the lac operon control. We find that the use of ALA and autoinduction leads to more consistent levels of holoprotein expression. To avoid the freezing of protein samples, we stored our purified enzymes in buffered 50% glycerol. Under these conditions, the flavohemoglobins retain their activity and characteristic UV-visible spectroscopic features for at least six months. Figure 3 shows a gel of our purified enzymes. At pH 6.5 and 37 °C, the NOD turnover number of Hmp (34 ± 2 s^−1^) is 1.5 fold higher that of gFlHb (23 ± 2 s^−1^), based on five separate experiments run on different days over a period of four months with three replicates each.

### 3.1. Nitric Oxide Dioxygenase Assays and Inhibition Studies

**Figure 4:**
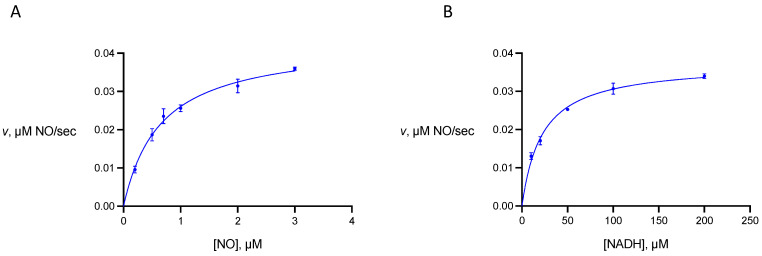
Substrate dependence of the nitric oxide dioxygenase activity of Giardia flavohemoglobin. (**A**) rate dependence on nitric oxide concentration. The concentration of NADH is held at 100 μM. (**B**) rate dependence on NADH concentration. The concentration of NO is held at 2 μM.

Michaelis constants of gFlHb towards NO and NADH were determined from rate measurements in which the concentrations of these substrates were varied (Figure 4). We find that for gFlHb *K*_mNO_ is 0.65 ± 0.09 μM and *K*_mNADH_ is 22 ± 4 μM. We did not determine the Michaelis constant of gFlHb for oxygen, but this has been reported as 22 ± 7 μM [7]. The comparable values for Hmp reported by Gardner and coworkers are 0.28 μM (*K*_mNO_), 4.8 μM (*K*_mNADH_), and approximately 100 μM (*K*_mO2_) at 37 °C and pH 7 [12].

**Figure 5:**
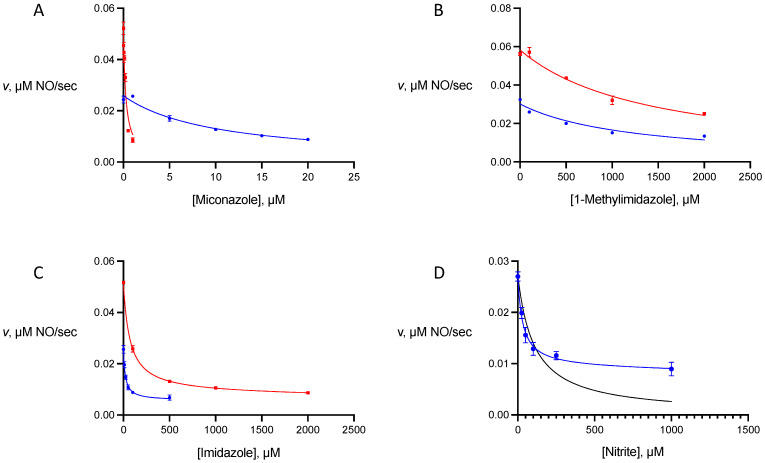
Plots of nitric oxide dioxygenase inhibition data. In all panels, data for gFlHb is denoted by the blue traces and Hmp by the red traces. Lines of best fit for miconazole (**A**) and 1-methylimidazole (**B**) were calculated by nonlinear regression for monophasic non-competitive inhibition. Values for imidazole (**C**) and nitrite (**D**) were calculated by nonlinear regression for biphasic non-competitive inhibition. For comparison, the black trace in (**D**) corresponds to a fit of the data to monophasic inhibition. Hmp nitric oxide dioxygenase activity is not significantly affected by nitrite and is not included.

To probe the sensitivity of NOD activities of gFlHb and Hmp to inhibition, we selected three compounds of different sizes that contain the imidazole ring, and nitrite. The imidazoles act as axial ligands to the heme, and one of the compounds we chose, miconazole, is known to be a potent non-competitive inhibitor of Hmp [10]. The other two compounds of this class that we examined were imidazole and 1-methylimidazole. Nitrite is also capable of acting as an axial ligand to heme proteins and its potential effects are of interest as it is a component of pathways involving reactive nitrogen species, acting as both a source of NO under acidic reducing conditions and an end product of uncatalyzed NO oxidation under aerobic conditions [13]. Plots of these experiments are presented in Figure 5 and calculated inhibition constants are presented in Table 1.

Inhibition by miconazole and 1-methylimidazole could be described by fitting the rate data to Equation (1) to obtain their inhibition constants. For both gFlHb and Hmp, the data for miconazole are consistent with competitive inhibition with a single mode of binding, and the *K*_i_ of 0.27 μM for Hmp is in reasonable agreement with that reported previously (0.08 μM), obtained at pH 7 [10]. The inhibition constant of miconazole towards gFlHb is nearly 40-fold higher (*K*_i_ = 10 μM) but it is still the most potent inhibitor tested for this enzyme. In this respect, its inhibition constant towards miconazole is similar to that measured for the YHb flavohemoglobin (~12 μM) of baker’s yeast [10]. Of the three imidazoles tested, 1-methylimidazole is the poorest inhibitor of both gFlHb and Hmp, with inhibition constants in both cases above 1 mM.

In contrast to miconazole and 1-methylimidazole, plots of the inhibition data for imidazole and nitrite gave poor fits to a single phase of non-competitive inhibition described by Equation (1) (see Figure 5D, black trace). This was also apparent from Dixon plots of 1/rate versus inhibitor concentration which deviated from the expected straight line for simple non-competitive inhibition. In these cases, nitrite oxide dioxygenase activity is sensitive to low concentrations of inhibitors, but residual activity persists even at a relatively high inhibitor concentration. For these inhibitors the rate data was fitted to a biphasic expression for non-competitive inhibition (Equation (2)).

(2) $$v=f*\frac{V_{max}}{\left(1+\frac{K_{mNO}}{s}\right)\left(1+\frac{\left[I\right]}{\;K_{i1}}\right)}+(1-f)\frac{V_{max}}{\left(1+\frac{K_{mNO}}{s}\right)\left(1+\frac{\left[I\right]}{K_{i2}}\right)}$$

Here *f* and 1 − *f* are the fraction of the observed inhibition contributed by the two phases that are described by inhibition constants *K*_i1_ and *K*_i2_, respectively. The inhibitor-sensitive phase accounts for 68–85% of the observed inhibition and inhibition constants can be calculated for this phase. However, the inhibition constants for the minority fraction that is far less sensitive to inhibition (15–32%) cannot be accurately determined with confidence from the data.

The biphasic inhibition of flavohemoglobins has been observed in cases where it is suspected that the enzyme was isolated with a fraction containing bound lipids at the active site, resulting in the enzyme being in a mixture of two states [10]. This is unlikely to be the case in our work, as lipids were absent from chloroform–methanol extracts of purified gFlHb and Hmp that were analyzed by thin-layer chromatography. Biphasic ligand binding has also been observed with small ligand binding to Hmp, and it was suggested that the ligand binding site could exist in two states [14]. This is supported by studies on gFlHb and Hmp by resonance-Raman spectroscopy using carbon monoxide as a probe for the binding of small ligands, which provided evidence for two binding modes denoted “closed” and “open”; the ligand makes strong polar contacts with conserved distal residues Tyr B10 and Gln E7 in the closed mode, while such interactions are absent in the open mode. This difference between ligand-binding conformational states seems to be of more significance for smaller ligands than for larger ones.

Resonance-Raman spectroscopy of purified gFlHb and Hmp shows that heme is five coordinate with no exogenous axial ligand [8,9], and structural characterization of Hmp by X-ray crystallography shows that this ligand binding site is obscured by the residues of helix E, with the side chain of conserved Leu E11 lying directly above the heme iron. While the distal face of flavohemoglobins can accommodate large ligands such as miconazole, this requires a large-scale movement of this helix to occur.

It is interesting that there is such a large difference in the ability of 1-methylimidazole and imidazole to act as inhibitors and in their binding profiles. A comparison of their data is revealing on several levels. Both are of comparable size and differ only by the presence of a methyl group in the former. Nonetheless, the effect is striking and influences not only binding strength but the mode of inhibition. The data for imidazole inhibition are clearly biphasic but those for 1-methylimidazole less so, and its data can be fit to a single inhibitor function. It seems that the boundary between the behaviour of a small ligand, with two possible binding modes, versus a large ligand with a single binding mode, is itself quite small. Even so, imidazole bound to the presumed closed conformation is a much more effective inhibitor than 1-methylimidazole. For both molecules, the movement of helix E required for ligand binding does not have to be as large as would be necessary to accommodate a large ligand such as miconazole; nonetheless, the presence of the additional methyl group of 1-methylimidazole likely causes steric clash that greatly weakens its ability to act as an inhibitor. This presumed steric effect occurs with both gFlHb and Hmp, which have similar inhibition constants towards each of these ligands.

In contrast to the similar behaviour of the two flavohemoglobins to these two smaller imidazoles was the difference in the effect of nitrite on the activities of gFlHb and Hmp. This ligand inhibits gFlHb at sub-millimolar concentrations (*K*_i_ = 32 μM) but has no significant effect on the activity of Hmp at concentrations up to 2 mM. To explore this further we performed optical titrations of both ferric gFlHb and Hmp with sodium nitrite. For gFlHb the binding constant *K*_D_ for nitrite was 180 ± 20 μM, while the optical differences of Hmp in the presence of nitrite were too small to permit any such calculation. It is also interesting to note that the binding constant of nitrite for gFlHb is larger than its inhibition constant, which indicates that nitrite inhibition of gFlHb involves more than one process. In addition to acting as a competitive inhibitor, it may act to impede either NO binding or more likely the departure of the nitrate product, which is of similar size and identical charge. That this is observed with gFlHb but not with Hmp may be evidence for different routes of product departure from the heme cofactor which is buried in the interior of the globin domain.

### 3.2. NADH Oxidase and Cytochrome c Reductase Activities

Although flavohemoglobins have high nitric oxide dioxygenase activity they will oxidize NADH in the absence of NO, and are able to reduce exogenous electron acceptors such as cytochrome *c* [15]. Our data on the NADH oxidase and cytochrome *c* reductase activity of gFlHb and Hmp are presented in Table 2.

As was observed with nitric oxide dioxygenase activity, the NADH oxidase activity of Hmp is higher than that of gFlHb. However, NADH oxidation seems to be more tightly coupled to NOD activity for gFlHb, as can be seen by comparing the ratio of these activities for the two enzymes: 0.014 for gFlHb and 0.026 for Hmp. Although modest, this difference may represent an evolutionary pressure for the Giardia enzyme to minimize non-specific electron transfer which could lead to reactive oxygen species, as Giardia lacks both superoxide dismutases and catalases, although it does have other enzymes that provide protection against superoxide and hydrogen peroxide [16]. Moreover, based on our observation that hydrogen peroxides more than double its NADH oxidase activity, it seems possible that gFlHb also plays such a role in the protection of Giardia from reactive oxygen species by acting as a hydrogen peroxide reductase. Hmp has such activity as well, although it is more efficient at reducing alkyl peroxides [17]. Our observations are consistent with the results of a transcriptional profiling experiment of Giardia trophozoites exposed to oxidative stress (O_2_ and hydrogen peroxide), which indicated that gFlHb was among the most highly up-regulated transcripts in response to these stressors, although the response also appeared to depend upon the Giardia genotype examined [18].

When provided with cytochrome *c* as an electron acceptor both flavohemoglobins have identical activities within experimental error. Electron transfer could occur either by direct interaction between the proteins to form a precursor docking complex, indirectly by superoxide generated from the flavohemoglobin, or a combination of both. Hmp has been reported to produce superoxide [19] which is a source of oxidative stress in *E. coli* [20] and we also find that a portion of its cytochrome *c* reductase activity (~17%) is inhibited by superoxide dismutase (SOD). Interestingly, we did not see any such effect of SOD on the cytochrome *c* reductase activity of gFlHb. Upon repeating this experiment, we found that 4% of this activity was inhibited by SOD. As noted above Giardia lacks superoxide dismutases and consequently its oxidoreductases may be under selective pressure to minimize the production of superoxide as a by-product.

## 4. Conclusions

The NOD activity of gFlHb is comparable to that of Hmp but differs in several respects, the most notable of which is the sensitivity of the former towards nitrite. The physiological significance of this is unknown, but we note that it is possible for organisms that inhabit the gastrointestinal tract to encounter nitrite in the dietary tract. Furthermore, although nitrite itself is not a reactive nitrogen species it is an end product of NO oxidation as well as a source of NO under reducing conditions. The NADH oxidase and cytochrome *c* reductase activities of gFlHb are consistent with the properties of an oxidoreductase from a microaerophilic protist that lacks conventional enzymes (catalase, superoxide dismutase) to provide protection from reactive oxygen species, and it is possible that gFlHb serves a dual role in protection from both nitric oxide and oxygen stressors.

## Figures and Tables

**Table 1:** Nitric oxide dioxygenase inhibition constants (*K*_i_) for flavohemoglobins calculated from the data presented in Figure 5. The parameter *f* corresponds to the fraction of inhibition (*f*) that can be accounted for with this *K*_i_. Nitrite did not significantly inhibit Hmp and its *K*_i_ was not determined (nd).

		gFlHb			Hmp	
Compound	*K*_i_ (μM)	*f*	TN, s^−1^	*K*_i_ (μM)	*f*	TN, s^−1^
Miconazole	10 ± 2	1	22 ± 1	0.27 ± 0.07	1	33 ± 3
1-methylimidazole	1200 ± 300	1	25 ± 1	1400 ± 250	1	38 ± 2
Imidazole	20 ± 6	0.78 ± 0.05	22 ± 1	69 ± 10	0.84 ± 0.02	33 ± 1
Nitrite	32 ± 12	0.68 ± 0.05	23 ± 1	nd	nd	nd

**Table 2:** NADH oxidase and cytochrome *c* reductase activities of flavohemoglobins. Where used, CuZn superoxide dismutase (SOD) was added to 5 U/mL.

	Specific Activity, s^−1^	
Assay	gFlHb	Hmp
NADH Oxidase	0.33 ± 0.09	0.87 ± 0.08
NADH Oxidase + 1 mM H_2_O_2_	0.7 ± 0.2	0.6 ± 0.1
Cytochrome *c* reductase	1.81 ± 0.06	1.8 ± 0.1
Cytochrome *c* reductase + SOD	1.8 ± 0.1	1.50 ± 0.06

## Data Availability

The data presented in this study are available on request from the corresponding author.

## References

[1] Ermler U., Siddiqui R.A., Cramm R., Friedrich B. (1995). Crystal structure of the flavohemoglobin from *Alcaligenes eutrophus* at 1.75 A resolution. EMBO J..

[2] Gardner P.R., Gardner A.M., Martin L.A., Salzman A.L. (1998). Nitric oxide dioxygenase: An enzymic function for flavohemoglobin. Proc. Natl. Acad. Sci. USA.

[3] Zhu H., Riggs A.F. (1992). Yeast flavohemoglobin is an ancient protein related to globins and a reductase family. Proc. Natl. Acad. Sci. USA.

[4] Iijima M., Shimizu H., Tanaka Y., Urushihara H. (2000). Identification and characterization of two flavohemoglobin genes in *Dictyostelium discoideum*. Cell Struct. Funct..

[5] Ioannidis N., Cooper C.E., Poole R.K. (1992). Spectroscopic studies on an oxygen-binding haemoglobin-like flavohaemoprotein from *Escherichia coli*. Biochem. J..

[6] Rafferty S., Luu B., March R.E., Yee J. (2010). Giardia lamblia encodes a functional flavohemoglobin. Biochem. Biophys. Res. Commun..

[7] Mastronicola D., Testa F., Forte E., Bordi E., Pucillo L.P., Sarti P., Giuffrè A. (2010). Flavohemoglobin and nitric oxide detoxification in the human protozoan parasite *Giardia intestinalis*. Biochem. Biophys. Res. Commun..

[8] Mukai M., Mills C.E., Poole R.K., Yeh S.R. (2001). Flavohemoglobin, a globin with a peroxidase-like catalytic site. J. Biol. Chem..

[9] Lukaszewicz B., McColl E., Yee J., Rafferty S., Couture M. (2017). Resonance Raman studies on the flavohemoglobin of the protist *Giardia intestinalis*: Evidence of a type I/II-peroxidase-like heme environment and roles of the active site distal residues. J. Biol. Inorg. Chem..

[10] Helmick R.A., Fletcher A.E., Gardner A.M., Gessner C.R., Hvitved A.N., Gustin M.C., Gardner P.R. (2005). Imidazole antibiotics inhibit the nitric oxide dioxygenase function of microbial flavohemoglobin. Antimicrob. Agents Chemother..

[11] Crowley E.L., Rafferty S.P. (2019). Review of lactose-driven auto-induction expression of isotope-labelled proteins. Protein Expr. Purif..

[12] Gardner A.M., Martin L.A., Gardner P.R., Dou Y., Olson J.S. (2000). Steady-state and transient kinetics of *Escherichia coli* nitric-oxide dioxygenase (flavohemoglobin). The B10 tyrosine hydroxyl is essential for dioxygen binding and catalysis. J. Biol. Chem..

[13] Ignarro L.J., Fukuto J.M., Griscavage J.M., Rogers N.E., Byrns R.E. (1993). Oxidation of nitric oxide in aqueous solution to nitrite but not nitrate: Comparison with enzymatically formed nitric oxide from L-arginine. Proc. Natl. Acad. Sci. USA.

[14] Ilari A., Bonamore A., Farina A., Johnson K.A., Boffi A. (2002). The X-ray structure of ferric *Escherichia coli* flavohemoglobin reveals an unexpected geometry of the distal heme pocket. J. Biol. Chem..

[15] Poole R.K., Rogers N.J., D’mello R.A.M., Hughes M.N., Orii Y. (1997). *Escherichia coli* flavohaemoglobin (Hmp) reduces cytochrome c and Fe(III)-hydroxamate K by electron transfer from NADH via FAD: Sensitivity of oxidoreductase activity to haem-bound dioxygen. Microbiology.

[16] Mastronicola D., Falabella M., Forte E., Testa F., Sarti P., Giuffrè A. (2016). Antioxidant defence systems in the protozoan pathogen *Giardia intestinalis*. Mol. Biochem. Parasitol..

[17] Bonamore A., Gentili P., Ilari A., Schininà M.E., Boffi A. (2003). *Escherichia coli* flavohemoglobin is an efficient alkylhydroperoxide reductase. J. Biol. Chem..

[18] Ma’ayeh S.Y., Knörr L., Svärd S.G. (2015). Transcriptional profiling of Giardia intestinalis in response to oxidative stress. Int. J. Parasitol..

[19] Wu G., Corker H., Orii Y., Poole R.K. (2004). *Escherichia coli* Hmp, an ‘oxygen-binding flavohaemoprotein’, produces superoxide anion and self-destructs. Arch. Microbiol..

[20] Membrillo-Hernández J., Ioannidis N., Poole R.K. (1996). The flavohaemoglobin (HMP) of *Escherichia coli* generates superoxide in vitro and causes oxidative stress in vivo. FEBS Lett..

